# Synthetic Data Enhancement and Network Compression Technology of Monocular Depth Estimation for Real-Time Autonomous Driving System

**DOI:** 10.3390/s24134205

**Published:** 2024-06-28

**Authors:** Woomin Jun, Jisang Yoo, Sungjin Lee

**Affiliations:** 1 Electronic Engineering, Dong Seoul University, Seongnam 13117, Republic of Korea; 2 Autonomous Driving Lab, Modulabs, Seoul 06252, Republic of Korea; 3 College of Electronics and Information, Kyung Hee University, 1732, Deogyeong-Daero, Giheung-gu, Yongin-si 17104, Republic of Korea

**Keywords:** autonomous driving, monocular depth estimation, absolute relative error, data augmentation, quantization, pruning

## Abstract

Accurate 3D image recognition, critical for autonomous driving safety, is shifting from the LIDAR-based point cloud to camera-based depth estimation technologies driven by cost considerations and the point cloud’s limitations in detecting distant small objects. This research aims to enhance MDE (Monocular Depth Estimation) using a single camera, offering extreme cost-effectiveness in acquiring 3D environmental data. In particular, this paper focuses on novel data augmentation methods designed to enhance the accuracy of MDE. Our research addresses the challenge of limited MDE data quantities by proposing the use of synthetic-based augmentation techniques: Mask, Mask-Scale, and CutFlip. The implementation of these synthetic-based data augmentation strategies has demonstrably enhanced the accuracy of MDE models by 4.0% compared to the original dataset. Furthermore, this study introduces the RMS (Real-time Monocular Depth Estimation configuration considering Resolution, Efficiency, and Latency) algorithm, designed for the optimization of neural networks to augment the performance of contemporary monocular depth estimation technologies through a three-step process. Initially, it selects a model based on minimum latency and REL criteria, followed by refining the model’s accuracy using various data augmentation techniques and loss functions. Finally, the refined model is compressed using quantization and pruning techniques to minimize its size for efficient on-device real-time applications. Experimental results from implementing the RMS algorithm indicated that, within the required latency and size constraints, the IEBins model exhibited the most accurate REL (absolute RELative error) performance, achieving a 0.0480 REL. Furthermore, the data augmentation combination of the original dataset with Flip, Mask, and CutFlip, alongside the *SigLoss* loss function, displayed the best REL performance, with a score of 0.0461. The network compression technique using FP16 was analyzed as the most effective, reducing the model size by 83.4% compared to the original while maintaining the least impact on REL performance and latency. Finally, the performance of the RMS algorithm was validated on the on-device autonomous driving platform, NVIDIA Jetson AGX Orin, through which optimal deployment strategies were derived for various applications and scenarios requiring autonomous driving technologies.

## 1. Introduction

Autonomous driving technology is one of the most extensively researched areas in recent times, owing to its wide applicability and significant impact across industries. Such vehicles are equipped with the capability to perceive and analyze their surrounding environment without a human driver, facilitating safe navigation to the designated destinations. However, one of the key challenges that these innovative technologies continue to face is the capability to accurately and swiftly comprehend and process three-dimensional space for real-time autonomous driving decisions [1,2]. Three-dimensional spatial perception plays a crucial role in autonomous driving systems, leading to research efforts that aim to perform depth estimation using expensive equipment such as LiDAR through sensor fusion techniques [3,4,5,6,7,8]. However, for effective implementation and widespread commercial application, it is preferable to perform depth estimation of objects and scenes quickly and cost-effectively using only a single camera [9,10]. This MDE (Monocular Depth Estimation) technique maximizes the safety and efficiency of autonomous vehicles by providing essential three-dimensional information needed to understand the surrounding vehicular environment, avoid obstacles, and plan safe routes [9,10]. In the field of MDE, a variety of methods have been developed [9,10,11]. However, these methods often encounter limitations in addressing real-world variability, such as complex lighting conditions, diverse meteorological circumstances, and the variety in objects and textures. However, these performance limitations can be substantially improved by leveraging rapidly advancing deep learning technologies if comprehensive datasets are available that include data for diverse scenarios, such as complex lighting conditions, varied meteorological conditions, textures of different objects, and advanced data augmentation techniques. In this study, we introduce synthetic-based data augmentation techniques that account for data diversity. Specifically, we propose a Mask method, which segments objects of interest from one image and synthesizes them onto another image. This approach is further enhanced with Mask-Scale, which involves resizing adjustments, and CutFlip, based on image flipping, to maximize the utilization of natural features and textures within existing datasets. Furthermore, we have derived optimal data augmentation strategies for contemporary MDE technologies by combining these techniques with other data augmentation methods previously suggested in various studies. Building on this, we propose the RMS algorithm, which integrates the latest MDE techniques, loss functions for MDE training, network compression methods, and system deployment considerations. This algorithm is designed to derive optimal application strategies tailored to specific autonomous driving applications.

The following section introduces the research related to the proposed techniques and outlines the major contributions. Section 2 details the technical aspects of the MDE methods. Section 4 describes the proposed data augmentation techniques, specifically the Mask, Mask-Scale, and CutFlip methods. Section 5 elaborates on the three operational stages of the proposed RMS algorithm. In Section 6, the performance of the proposed techniques is validated, and Section 7 concludes the discussion.

## 2. Related Work

Prior to the advent of deep learning, early research in MDE primarily revolved around depth-cue-based approaches [12,13,14]. Study [12] utilized an approach based on the vanishing point, study [13] focused on depth perception derived from focus and defocus techniques, and study [14] employed a shadow-based approach. However, these studies were constrained by their ability to perform MDE under limited conditions, rendering them inadequate for application in real-world settings with diverse variations.

With the advent and progression of deep learning [15,16,17], research in the field of MDE also began to incorporate deep learning methodologies [18]. This approach is characterized by an encoder–decoder structure that receives RGB input and produces depth maps. Subsequently, numerous studies [18,19,20,21,22,23,24,25] emerged, adopting a similar encoder–decoder framework. Further advancements were made as research [26,27,28] explored the generation of depth maps based on probabilistic combinations of sequential images using CRFs (Conditional Random Fields) applied to the output feature maps of the encoder. In study [26], depth maps were derived by extracting feature maps of various sizes from consecutive images and combining them using an attention-based mechanism. Additionally, the application of CRFs was diversified, with study [29] implementing multiple cascade CRFs, study [27] using continuous CRFs, study [28] applying hierarchical CRFs, and study [30] employing FC-CRFs (Fully Connected CRFs) for performing MDE.

However, the application of supervised learning to MDE incurs high data-labeling costs. To mitigate this, attempts have been made to employ unsupervised learning methodologies [31,32,33,34,35,36]. These studies, predominantly based on image reconstruction techniques, stereo matching, and depth extraction through camera pose estimation from consecutive video frames, introduce additional complexities without achieving significant advancements in accuracy. Meanwhile, as an alternative approach to overcoming the issue of insufficient data in MDE, several research attempts have been made to generate variant data to supplement the scarce training dataset. In studies [37,38,39,40,41,42,43,44,45,46,47], attempts were made to augment and utilize existing data through various methods such as data augmentation techniques, style transfer, and data synthesis. In the studies by [48,49,50], the use of copy-and-paste-based data augmentation techniques was explored to enhance performance in various tasks, specifically object detection and segmentation. This approach involves integrating elements from one image into another to enrich the dataset and improve the robustness of the models trained for these applications. Similarly, the study by [37] explored data augmentation for MDE in their CutDepth approach by pasting rectangular regions from one image onto the original image. This method was analyzed to enhance the REL performance by approximately 1.5%, demonstrating its efficacy in improving depth estimation accuracy. Based on the CutDepth framework, various composite techniques have emerged, specifically vertical orientation and perpendicular orientation techniques, referred to as vertical CutDepth [51] and perpendicular CutDepth [52], respectively. These methods have demonstrated performance improvements comparable to those achieved with the original CutDepth approach. In studies [38,39,40,41,42,43,44,45,46,47], classical methods involving noise, brightness, contrast adjustments, and multi-scale and geometric transformations were applied to MDE techniques to enhance accuracy. These approaches resulted in achieving an REL performance metric of 0.112 on the KITTI dataset, according to the analysis. The study [53] analyzed the performance of a simple encoder–decoder-based MDE model by applying data augmentation techniques such as scale, rotation, color jitter, color normalization, and flips, utilizing geometric variations and filtering methods. The application of these techniques achieved an REL performance of 0.066 on the KITTI dataset, as analyzed. Several studies [54,55,56,57] have utilized Generative Adversarial Network (GAN) technology to generate data or perform style transformations for data augmentation purposes. In particular, research by [55,56,57] focused on implementing data augmentation techniques tailored to various weather conditions to enhance robust performance. Additionally, the study [58] explored the creation of image data and corresponding depth labels within a virtual environment for use as data resources. The study [59] introduced reliable data augmentation that minimizes the loss between disparity maps generated by the original and augmented images, enhancing image robustness in predicting color fluctuations. Similarly, in the study by [60], an attempt was made to enhance the performance of depth estimation by applying augmentation at the feature representation level derived from the results of an image encoder. Research [61] implemented a data augmentation technique based on supervisory loss, improving depth at occluded edges and image boundaries while making the model more resilient to changes in illumination and image noise. The study [62] generated multi-perspective views and corresponding depth maps based on NeRFs (Neural Radiance Fields), utilizing interpolated- and angle-variation-based data augmentation methods, and conducted performance evaluations for AdaBins [63], DepthFormer [64], and BinsFormer [65].

In parallel, substantial research has been undertaken to enhance MDE technologies for dependable and real-time performance on devices with limited resources, such as autonomous vehicles, robotics, and embedded systems. These advancements focus on model compression, lightweight architectures, and acceleration techniques, which are typically grouped into pruning [66,67], the development of efficient architectures [68,69,70], the application of knowledge distillation [71,72], and real-time operation [66,67,68,69,70,71,72,73]. This body of work aims to refine MDE functionality to suit the computational constraints of various hardware platforms, enhancing operational efficiency across multiple application domains. In the studies by [66,67], pruning techniques and similar methods were explored with the objective of energy conservation through targeted weight training approaches. These methods focus on reducing the computational demands of models by selectively pruning less important network weights, thereby enhancing energy efficiency during operations. In the study by [68], a lightweight design approach for the encoder–decoder network in MDE was addressed. Specifically, the research utilized MobileNet to reduce the weight of the encoder–decoder structure. By replicating this streamlined architecture twice, the study aimed to mitigate the loss of accuracy typically associated with reductions in model complexity. In the study by [69], visual domain adaptation was employed to minimize accuracy degradation within a lightweight network structure based on MobileNet. The research by [70] aimed to enhance prediction accuracy through a lightweight design that incorporates elements from the biological visual system and self-attention mechanisms. Meanwhile, the studies [71,72] explored the use of KD (Knowledge Distillation) to streamline the traditional encoder–decoder architecture in MDE. However, despite these technologies’ ability to significantly reduce latency—by up to a factor of ten—accuracy degradation remains a substantial limitation. Finally, the technologies for the real-time operation of autonomous driving computations can utilize the previously described model lightweight techniques, namely pruning [66,67], efficient architecture [68,69,70], and knowledge distillation [71,72]. However, while these model lightweight techniques can reduce the size of the model, they do not always decrease operational latency because they may require additional computations for the model operation. Therefore, it is essential to deploy and analyze the performance on actual embedded devices to verify their effectiveness.

Despite advancements in various data augmentation techniques, the current REL performance still presents limitations for commercial deployment. The reason for this is that the proposed data augmentation techniques do not necessarily guarantee performance improvements. Specifically, data augmentation methods based on color filters (e.g., color jitter, color normalization, brightness control, contrast control) tend to exhibit variability in performance enhancement compared to geometric variation techniques. Moreover, the performance can vary depending on the MDE model, making it challenging to ensure performance improvements in recent MDE models.

Consequently, there is a demand for developing geometric-variation-based data augmentation techniques that can consistently yield performance enhancements across all MDE technologies. Furthermore, it is essential to identify combinations of data augmentation techniques that can effectively enhance the performance of recent MDE models through the integrated use of traditional augmentation methods.

In this research, we have developed data augmentation techniques based on geometric variations, specifically Mask, Mask-Scale, and CutFlip, that can reliably enhance the accuracy of MDE. We particularly investigated the optimal combinations of these techniques with traditional data augmentation methods such as scaling, rotation, translation, noise, and brightness control by analyzing their performance synergies. Additionally, we conducted experimental analyses to determine the most effective strategies for maximizing MDE accuracy across various loss functions and optimized these strategies for operational latency and memory efficiency through network lightweighting techniques. This approach is applicable to supervised, unsupervised, and semi-supervised learning, offering a viable method for enhancing the accuracy of monocular depth prediction in robotic and autonomous driving environments.

The contributions of this paper are summarized as follows:

- Proposal of Novel Synthetic-Based Data Augmentation Techniques for MDE Performance Enhancement: This paper proposes new synthetic-based data augmentation methods, such as Mask, Mask-Scale, and CutFlip, to improve monocular depth estimation performance and derive the optimal combination of data augmentation techniques.
- Proposal of Network Compression Methods for Enhanced Efficiency in Real-Time MDE: Strategies to minimize the size and operational time of real-time monocular depth estimation models through quantization and pruning techniques are suggested.
- Optimal Application Strategies for Autonomous Driving Systems Considering Performance: This paper presents the RMS algorithm, an optimal strategy tailored for commercial autonomous driving applications, taking into account the current MDE performances on high-end servers and on-device systems. This strategic approach is designed to harness the capabilities of different deployment environments effectively.

## 3. Technical Details for Performance Analysis and Enhancement of MDE

### 3.1. Base Model

For the RMS algorithm, MonoDepth [20], DepthFormer [64], and IEBins [65] are considered as base model options for performance analysis and real-time configuration.

Firstly, MonoDepth [20] follows a fundamental encoder–decoder structure. The encoder, based on ResNet, extracts features to learn visual characteristics. The decoder, comprising convolution and upsampling operations, fuses features from the encoder to restore resolution and predicts high-resolution depth maps. Here, the encoder can be diversified using techniques such as FPNs (Feature Pyramid Networks) [74], Bi-FPNs (Bidirectional Feature Pyramid Networks) [75], and PFPNs (Panoptic Feature Pyramid Networks) [76].

DepthFormer [64] also adheres to an encoder–decoder structure but introduces the HAHI (Hierarchical Aggregation and Heterogeneous Interaction) module between the encoder and decoder to enhance the model’s performance. The HAHI module models interactions and relations between features *F* and *G* obtained from the transformer and convolution branches.

Lastly, IEBins [65] also adheres to an encoder–decoder structure based on skip connections. The encoder utilizes Swin Transformer as its backbone and is composed of a four-level feature pyramid. Each skip connection links pyramid features to the decoding phase. The decoder employs three CRF modules to capture long-range correlations and uses an iterative optimizer to extract context features, which are then fed into the GRU hidden state. Ultimately, the depth map is derived from the linear combination of the probability distribution outputs of the three stages.

### 3.2. Loss Function

In the context of monocular depth estimation, loss functions are pivotal for quantifying the disparity between actual depth values and those predicted by the model. For this purpose, *SigLoss* (scale-invariant gradient loss) $L_{Sig}$ and *BerhuLoss* $L_{Berhu}$ [77] were employed as loss functions, with the more effective loss function value being selected and utilized based on experimental outcomes.

First of all, the formulas of *SigLoss* [78] are as follows:

(1) $$\begin{array}{ccc} d_{i} & = & \log\left(d\right)-\log\left(d^{*}\right),\\ L_{SigLoss} & = & \frac{1}{T}\sum_{i}d_{i}^{2}-\frac{1}{T^{2}}{\left(\sum_{i}d_{i}\right)}^{2} \end{array}$$

where *d* represents the predicted value, $d^{*}$ the true depth value, and *c* the threshold.

The *SigLoss* is designed to reduce dependency on the absolute values of depth prediction. Most depth estimation methods heavily rely on absolute depth values, which can often be inaccurately estimated. *SigLoss* focuses on the relative relationships or gradients between log-based depth values within an image, ensuring that the overall structure of the predicted depth map remains similar to that of the original depth map. This approach shifts the focus from absolute depth values to maintaining structural integrity in depth estimation.

Next, *BerhuLoss* is defined as follows:

(2) $$\begin{array}{cc} L_{Berhu}\left(d,d^{*}\right) & =\left\{\begin{array}{cc} |d-d^{*}| & ,\;\mathrm{if}\;|d-d^{*}|\;\leq c,\\ \frac{|d-d^{*}{|}^{2}+c^{2}}{2c} & ,\;\mathrm{if}\;|d-d^{*}|\;>c. \end{array}\right. \end{array}$$

*BerhuLoss* operates such that when the error is less than or equal to a threshold *c*, it directly uses the error $|d-d^{*}|$. However, for errors exceeding the threshold *c*—indicating that the prediction error surpasses the set limit—the formula adds a constant $c^{2}$ and divides by 2c, thus aggressively eliminating outliers while still allowing for some error. This approach ensures that the model is not overly sensitive to large errors, facilitating stable learning.

### 3.3. Network Compression

Model quantization is a technique extensively utilized to condense and expedite the inference phase in deep learning systems. This technique involves compressing network weights by reducing the bit representation, typically from 32 bits to a lower bit rate. Consequently, quantization constrains the dynamic range and precision of bit representation but offers the benefit of significantly diminishing the overall network weight size, proportional to the reduction in bits.

These quantization techniques can be categorized based on their approach into Quantization-Aware Training (QAT) and Post-Training Quantization (PTQ). Further, PTQ can be subdivided into three distinct methods depending on the degree and manner of compression: Baseline Quantization (BLQ), Full-Integer Quantization (FIQ), and Float 16 Quantization (F16), as outlined in [79].

According to previous research [79], among various quantization techniques, only FP16 uniquely offers the benefit of reducing size without compromising latency and accuracy. Consequently, this paper focuses on the FP16 method as the quantization technique of choice.

On the other hand, the pruning technique in deep learning operations involves retaining weights that exceed a certain threshold value and setting the remaining weights to zero. This approach typically involves sorting some of the weights based on their absolute values and then zeroing out the smallest ones until a specific level of sparsity is achieved, as outlined in [80]. In this study, we incrementally increased the number of weights set to zero in the CNN models over 60–70 iterations to optimize accuracy.

## 4. Proposed Data Augmentation Techniques for MDE

**Figure 1:**
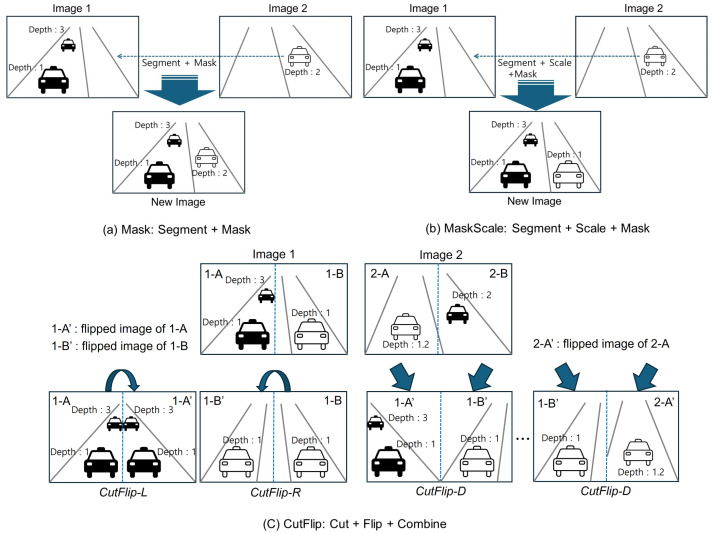
Illustrative diagrams of Mask, Mask-Scale, and CutFlip: 1-A represents the left part of image 1, 1-A’ is the flipped version of 1-A, 2-A represents the left part of image 2, 2-A’ is the flipped version of 2-A, 1-B represents the right part of image 1, 1-B’ is the flipped version of 1-B, 2-B represents the right part of image 2, 2-B’ is the flipped version of 2-B.

**Figure 2:**
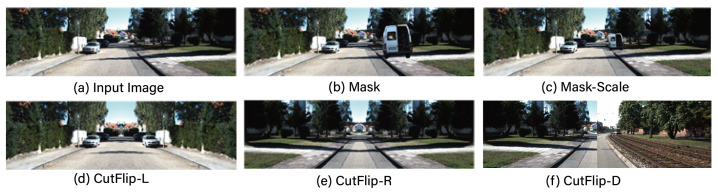
Application examples of Mask, Mask-Scale and variations of CutFlip.

MDE faces a significant challenge due to the high costs associated with data acquisition and labeling, resulting in substantially fewer training data compared to other image recognition tasks. Consequently, the application of data augmentation is essential to compensate for the insufficient quantities of training data for MDE. However, traditional data augmentation techniques such as flipping, scaling, noise addition, brightness adjustment, and rotation encounter limitations in enhancing performance due to a lack of data diversity. In this section, we propose techniques that go beyond variations within a single image, introducing methods that synthesize data across multiple images, namely Mask, Mask-Scale, and CutFlip. Table 1 outlines the definitions of these techniques, Figure 1 presents their illustrative diagrams, and Figure 2 depicts examples of applying Mask, Mask-Scale, and CutFlip.

In this study, we conducted experiments applying the proposed data augmentation techniques on the KITTI dataset as the original data source [81]. The primary reason for utilizing the KITTI dataset is that it not only provides depth map data for MDE but also encompasses classes such as cars, pedestrians, bicycles, and people, which are crucial for autonomous driving in outdoor environments.

When augmenting data using the Mask, Mask-Scale, and CutFlip techniques, the corresponding depth map should be synthesized in the same manner as the altered image. As described in Figure 1a, when augmenting data with Mask, the depth map information is masked in the depth map exactly as the masked object’s depth information and location. Conversely, as explained in Figure 1b, when applying Mask-Scale, the depth map information is adjusted inversely proportional to the scale ratio. For CutFlip-L and CutFlip-R applications, as illustrated in Figure 1c, the depth map from either the left or right side is directly copied to the opposite side. CutFlip-D combines several images by selectively applying Flip to the left or right images, and the corresponding depth maps are combined and structured similarly.

The traditional data augmentation methods mentioned above, namely flip, scale, noise, brightness and rotation, artificially create a variety of environmental changes that could be encountered in real driving scenarios. This integration during the training process enables a better REL performance in actual test datasets and enhances the model’s generalization capability. However, these methods face performance limitations in enhancing depth prediction based on object information due to insufficient variability and the scarcity of object data themselves caused by class imbalance. Conversely, the synthetic-based Mask method proposed in this paper employs segmentation techniques to extract objects from different images and synthesizes them onto the base image, including their depth information. This approach addresses the lack of high-quality depth prediction data based on object information, thereby contributing to an enhanced performance. Furthermore, the Mask-Scale data augmentation method overcomes a limitation of the Mask method, which is restricted to augmenting data based on the existing size of objects and their corresponding depth information in the original image. By proportionally varying the size of the objects and their depth information during augmentation, the Mask-Scale method enables the synthesis and augmentation of not only the objects themselves but also their associated depth information. Lastly, CutFlip represents one of the most efficient and straightforward methods for data augmentation. Unlike traditional data augmentation techniques, it possesses photorealistic qualities that closely mimic actual data, thereby significantly enabling the enhancement of REL performance during real testing scenarios.

## 5. Optimal Configuration Process of MDE According to Application

### 5.1. Overall Configuration Flow

**Figure 3:**
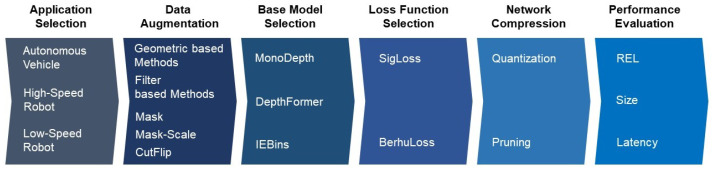
The overall operational process for optimal configuration of monocular depth estimation according to application.

The workflow of the entire system for optimizing MDE settings according to the application is depicted in Figure 3. Initially, an autonomous driving application suitable for MDE is selected. Since each autonomous driving application possesses distinct characteristics and associated performance requirements, this selection is prioritized to subsequently optimize the overall MDE system settings. Considered applications include autonomous vehicles, which must meet high levels of real-time performance, accuracy, and memory requirements of embedded systems due to the necessity to drive at high speeds while ensuring safety. In the realm of autonomous robots, these can be classified into high-speed and low-speed robots based on their operational velocities. High-speed robots, used for high-speed outdoor delivery purposes, require high mobility not unlike autonomous vehicles and must handle a variety of data variability and stringent performance requirements. Conversely, low-speed robots are utilized for indoor delivery at slower speeds, where environmental variables are relatively consistent and minimal, thereby influencing their performance requirements accordingly.

Subsequently, data augmentation is utilized to acquire a more diverse dataset. In reality, datasets sufficient for MDE are not adequately available, which leads to overfitting issues when training neural-network-based MDE models, thereby capping the potential for accuracy improvement. To address this, a combination of geometric-based methods such as rotation, flipping, and scaling, alongside filter-based methods like brightness control and noise addition and the proposed Mask, Mask-Scale, and CutFlip techniques, are employed to enhance dataset diversity.

The base model is then determined and configured. For this purpose, models based on the encoder–decoder structure, such as MonoDepth [20], DepthFormer [64], and IEBins [65], are established as the basic options for proceeding.

Following this, the loss functions are configured to achieve higher REL values. This step is critical as various functions can significantly impact the training accuracy of models trained on identical datasets and model architectures. Therefore, a range of functions are tested to determine the most effective ones.

The next phase involves exploring the potential for network compression. This is carried out not solely for enhancing REL accuracy but primarily to improve operational efficiency in embedded systems.

Finally, the completed model undergoes a comprehensive performance analysis. This evaluation is conducted from three perspectives: accuracy, as measured by the REL metric; efficiency, determined using the size metric; and real-time capability, assessed via the latency metric.

### 5.2. RMS Algorithm

**Figure 4:**
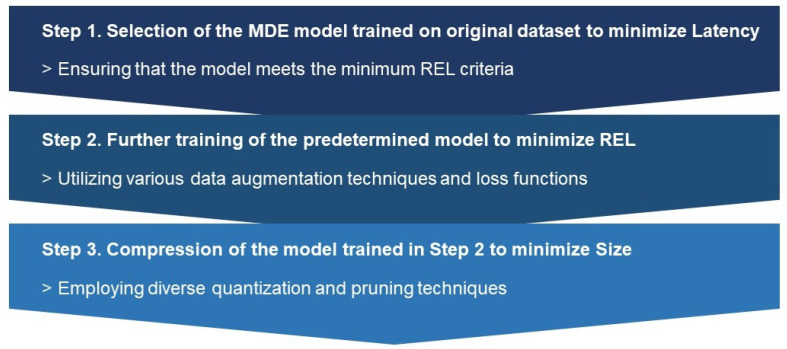
RMS algorithm for optimal configuration of monocular depth estimation.

A comprehensive greedy search for all possible configurations, as mentioned above, would be time-consuming and resource-intensive. To enable efficient implementation, strategic and systematic approaches to configuring these factors are necessary. For this goal, our research introduces the RMS (Real-time configuration of Monocular Depth Estimation considering REL, Size, and Speed) algorithm, structured in three steps as illustrated in Figure 4. This approach streamlines the process of determining the optimal configuration, balancing critical factors such as accuracy, model size, and latency, for effective monocular depth estimation in real-time applications.

For the elucidation of our algorithm, parameters are defined as depicted in Table 2.

#### 5.2.1. Selection of the MDE Model Trained on Original Dataset to Minimize Latency

Based on the original dataset and the loss function *SigLoss*, we select the MDE model $B^{*}$ that minimizes latency while satisfying the minimum REL condition, as determined by Equation (3). The candidate MDE models considered include MonoDepth [20], DepthFormer [64], and IEBins [65], though the inclusion of other models with demonstrated superior performance remains a feasible option.

(3) $$\begin{array}{ccc} B^{*} & = & \arg\min_{B}\left\{LAT\left(W_{B}\right)\right\}\\ \mathrm{Subject}\;\mathrm{To}\; & & REL(W_{B},D,L)\leq \gamma ,\\ & & B\in \{\mathrm{MonoDepth},\mathrm{DepthFormer},\mathrm{IEBins}\},\\ & & D\subset \{\mathrm{Original}\},\;\;\;L\in \{\mathrm{SigLoss}\}. \end{array}$$

In this framework, the minimum REL threshold γ is a critical parameter that balances the trade-off between the accuracy and latency performance of MDE. A lower REL threshold might lead to the absence of feasible solutions or unsatisfactory latency outcomes. On the other hand, a higher REL threshold broadens the feasible solution space, possibly yielding acceptable latency results, but at the risk of compromising MDE accuracy, which could negatively impact the safety of autonomous driving. Therefore, it necessitates a comprehensive evaluation, taking into account the intended level of autonomous driving and the given hardware specifications.

#### 5.2.2. Further Training of the Predetermined Model to Minimize REL

As shown in Equation (4), step 2 involves additional training to minimize the REL based on the base model selected in step 1. This step aims to optimize the weights of the base model using a variety of data augmentation techniques and loss function options. The dataset D includes Original, Flip, Rotation, Noise, Brightness, Mask, Mask-Scale, CutFlip, and their combinations. The loss functions employed include options of *SigLoss* and *BerhuLoss* for training. While all currently advantageous data augmentation methods and loss functions were utilized, the inclusion of alternative methods into this set allows for the expansion and application of the RMS algorithm.

(4) $$\begin{array}{ccc} W_{B^{*}}^{*},D^{*},L^{*} & = & \arg\min_{W_{B^{*}},D,L}\left\{REL\left(W_{B^{*}},D,L\right)\right\},\\ & & D\subset \{\mathrm{Original},\;\mathrm{Flip},\;\mathrm{Rotation},\;\mathrm{Mask},\;\mathrm{Mask}-\mathrm{Scale},\;\mathrm{CutFlip}\},\\ & & L\in \{\mathrm{SigLoss},\mathrm{BerhuLoss}\}. \end{array}$$

#### 5.2.3. Compression of the Model Trained in Step 2 to Minimize Size

Step 3 involves compressing the base model weights $W_{B^{*}}^{*}$, trained for minimizing latency and REL in steps 1 and 2, based on Equation (5). For network compression, methods such as FP32 (no quantization), FP16 (Float Point 16 quantization) and PRN (Pruning) are considered [79]. The objective is to perform network lightening within a range that maintains or minimally impacts the previously achieved REL and latency performance.

(5) $$\begin{array}{ccc} Q^{*} & = & \arg\min_{Q}\left\{SIZE\left(\Phi \left(Q,W_{B^{*}}^{*}\right)\right)\right\},\\ \mathrm{Subject}\;\mathrm{To}\; & & REL(\Phi \left(Q,W_{B^{*}}^{*}\right),D^{*},L^{*})\leq \gamma ,\\ & & LAT(\Phi \left(Q,W_{B^{*}}^{*}\right))\leq \tau ,\\ & & Q\in \{\mathrm{FP}32,\;\mathrm{FP}16,\;\mathrm{PRN}\}. \end{array}$$

## 6. Simulation Results

### 6.1. Data Augmentation and Model Selection Strategies

The experiments were primarily focused on validating the real-time feasibility of MDE through the RMS algorithm, improving the accuracy in terms of REL performance, and verifying the model size reduction. For the experimental setup, training was conducted on a single NVIDIA H100 GPU, while testing was performed on a dual RTX 4090 NVIDIA GPU setup, with programming and result analysis carried out using the Pytorch framework. The NVIDIA GPU 4090 RTX [82] used for inference offers an AI computation performance of 1321 TOPS and requires approximately 850 W of power. The dataset utilized for the experiments was based on the KITTI database, using 72,084 pairs of original RGB images, supplemented by an additional 72,084 pairs per each applied augmentation technique. Training was executed over 20 epochs with a batch size of 4 to evaluate performance. For the implementation of the RMS algorithm, detailed parameters were set, with γ configured to 0.05 and τ established at 100 ms.

Table 3 and Table 4 presents the performance metrics derived for steps 1 and 2 of the RMS algorithm. To determine the optimal base model for step 1, the performance in terms of REL and latency was demonstrated for MonoDepth, DepthFormer, and IEBins models, which were trained using the original dataset. Furthermore, for the optimization of additional training in step 2, the REL and latency performances obtained after subjecting these base models to various data augmentation techniques and different loss function configurations were also presented. Table 4 illustrates the latency and size performance values of the MDE base models, which are evaluated for steps 1 and 2 of the RMS algorithm.

As discernible from Table 3, the IEBins model is identified as the base model with the most commendable REL performance. Notably, IEBins is the only technology that satisfies the REL performance threshold, set at γ=0.05. Additionally, as evidenced in Table 4, it can be observed that IEBins exhibits a better performance compared to DepthFormer in terms of latency. Therefore, selecting IEBins as the base model from the options explored in this study is the most judicious choice, denoted as $B^{*}=\mathrm{IEBins}$. Step 2 of the RMS algorithm involves optimizing the REL performance through additional training of the identified optimal base model, IEBins, using an augmented dataset and a loss function. As evidenced in Table 3, the outcome of further training with various data augmentation combinations reveals that data augmentation significantly enhances REL performance. Specifically, the Ori + Flip + Mask + CutFlip combination yields the most exemplary result among all experimental sets, with a metric of 0.0461. This figure is an improvement of 0.0019 over the 0.0480 result achieved from training on the original dataset, translating to an approximate enhancement of $4.0\%=\frac{0.0019}{0.0480}$. The combinations of Ori + Flip + Mask + CutFlip, as well as Ori + Flip + Mask, Ori + Flip + Mask + Rotation + CutFlip, Ori. + Mask + Mask-Scale + CF, and Ori. + Scaling + Rotation + Translate + Noise + Flip + Mask + Mask-Scale + CF that incorporate the use of Mask, also demonstrate similarly excellent performance. This indicates that the proposed Mask-based data augmentation combinations are highly effective in enhancing the REL performance of MDE.

In the experimental results for data augmentation, it is notable that not all data augmentation techniques contribute to performance enhancement. Specifically, combinations such as Noise with Brightness, Scale with Brightness, and Flip with Noise showed a decrease in REL performance. Furthermore, the application outcomes of these data augmentation techniques demonstrated similar results not only in IEBins but also in MonoDepth and DepthFormer. This indicates that selective application of datasets through various experiments is essential for optimizing MDE performance.

**Figure 5:**
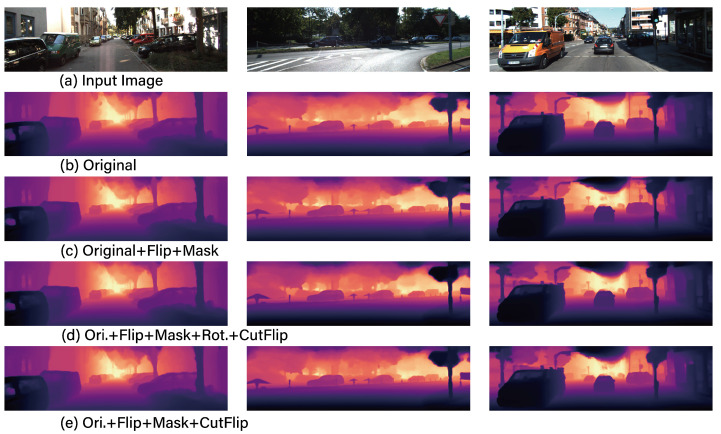
The example inference results of IEBins according to data augmentation technique combinations.

**Figure 6:**
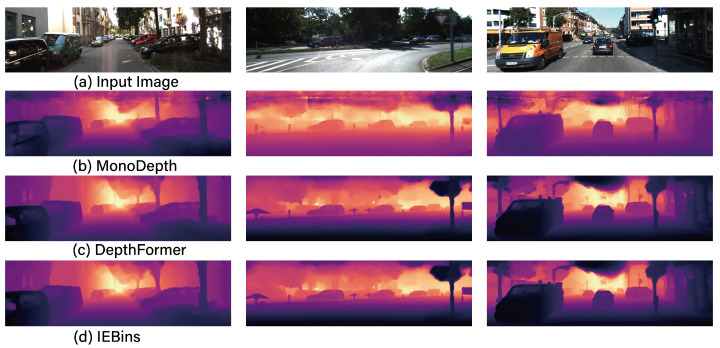
The comparison of results for MonoDepth, DepthFormer, and IEBins when applying the best-performing data augmentation combination of Ori + Flip + Mask + CutFlip and the *SigLoss* loss function.

Figure 5 displays exemplary result images from the combinations of data augmentation that have been additionally trained on the IEBins model. As observed in Figure 5, the depth predictions made using the Original + Flip + Mask + CutFlip dataset distinctly depict the presence and form of distant signs, aspects that were not discernible in the depth predicted using the original dataset. Figure 6 illustrates the results of the base models MonoDepth, DepthFormer, and IEBins when trained with the best data augmentation combination: Ori + Flip + Mask + CutFlip. As demonstrated in Figure 6, it is evident that IEBins more distinctly identifies shapes compared to MonoDepth and DepthFormer. Finally, it is observed that the *SigLoss* loss function demonstrates superior performance compared to the *BerhuLoss*. This implies that, for MDE problems, a log-difference-based *SigLoss* is more effective than a Euclidean difference-based *BerhuLoss*.

**Remark** **1.** *Mask-based data augmentation techniques are effective in enhancing the REL of MDE. Furthermore, the optimal data augmentation strategy for MDE is identified as the combination of Ori + Flip + Mask + CutFlip.*

**Remark** **2.** *For enhancing the REL, size, and latency performance in MDE, SigLoss is found to be more suitable than BerhuLoss.*

### 6.2. Network Compression Strategy

**Figure 7:**
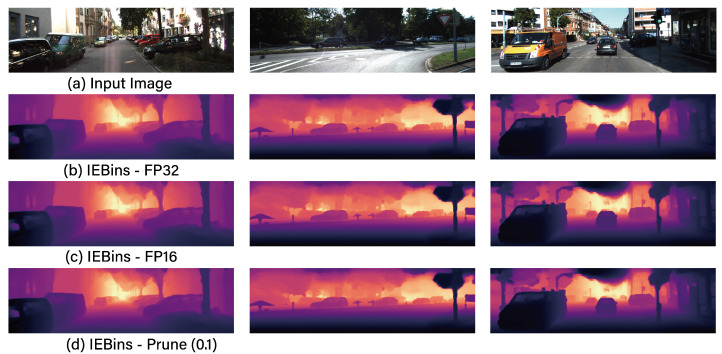
The example inference results for the IEBins model with the best performance based on network compression techniques.

Proceeding to step 3 of the RMS algorithm, the focus shifts to network compression. Table 5 showcases the results of compressing the IEBins network utilizing quantization and pruning techniques as delineated in Equation (5). As Table 5 indicates, the FP16 format achieves the highest compression rate at 83.4% $=1-\frac{0.54}{3.3}$ while simultaneously maintaining a robust REL and latency performance. In the context of pruning, the compression rate varies according to the adjustment in the ratio, meaning that setting a higher ratio results in a greater compression rate. However, considering the REL threshold of γ=0.05, the feasible range for the ratio is between 0.05 and 0.1. Given that the size and latency metrics for FP16 are superior to those achieved through pruning, it is evident that FP16 represents the most effective network compression method. Figure 7 exhibits example image results of the IEBins model corresponding to various network compression techniques.

**Remark** **3.** *The FP16-based quantization technique exhibits the most effective network compression performance, along with the best REL, size, and latency metrics.*

### 6.3. On-Device Strategy

Finally, the optimized models, MonoDepth, DepthFormer, and IEBins, which were trained and compressed under the best data augmentation combinations determined by the RMS algorithm, were deployed on the on-device AI platform NVIDIA Jetson AGX Orin [83] for performance analysis. The NVIDIA Jetson AGX Orin features a 2048-core GPU with 64 Tensor Cores and delivers up to 275 TOPS of AI computation performance, with power consumption settings ranging from 15 W to 75 W. Table 6 analyzes the latency performance of these models under 50W and 30W power modes of NVIDIA Jetson AGX Orin. Table 7 displays the size of each model following network compression by the RMS algorithm.

In the experimental results, it was observed that the NVIDIA AGX Orin platform demonstrated an increase in latency by approximately 8.75 times (821/93.8) under a 50 W setting and about 20.7 times (1942/93.8) under a 30 W setting compared to the traditional 4090 GPU. Additionally, power consumption decreased by 94% ($1-\frac{50}{850}$) at 50 W and by 96% ($1-\frac{30}{850}$) at 30 W. This results in significant power reduction, yet it also leads to considerable increases in latency, which can be observed through the experimental data. Consequently, it becomes evident that such settings are impractical for high-speed autonomous vehicles where real-time processing is critical. If the application is intended for more dynamic outdoor environments, it becomes evident that a distributed computing approach utilizing multiple NVIDIA Jetson AGX Orin units is necessary. Furthermore, as evidenced in Table 6, MonoDepth exhibits the lowest latency compared to other models, DepthFormer and IEBins. According to Table 3 and Table 7, although MonoDepth has the highest REL accuracy performance at 0.0610, which is less accurate compared to DepthFormer at 0.0510 and IEBins at 0.0461, it demonstrates a clear advantage in terms of memory size and latency. This suggests that MonoDepth is a suitable choice for indoor navigation robots operating in static environmental conditions.

Conversely, IEBins and DepthFormer, which show better REL performance as per Table 3, exhibit significantly poorer latency in low-power on-device environments. Therefore, employing these models in low-power on-device setups in actual autonomous driving vehicles appears to be impractical; they are more suited for low-speed outdoor or indoor navigation robots. If deploying MDE in an autonomous vehicle, it is recommended to use server-grade GPUs in environments where the power supply is stable in order to ensure effective performance.

Finally, DepthFormer shows inferior performance in both speed and accuracy compared to IEBins, as shown in Table 3 and Table 6. Hence, for all autonomous driving applications, using IEBins over DepthFormer is recommended.

## 7. Conclusions

This study investigates methods for optimizing real-time performance in monocular depth prediction within autonomous driving systems equipped with limited datasets and relying on a single camera for environmental depth perception. To achieve this, we propose a three-stage RMS algorithm experiment to optimize performance indicators in accuracy (REL), operational speed (latency), and memory size. In the first stage, a base model that satisfies REL performance criteria while minimizing latency is selected. Encoder–decoder-based models such as MonoDepth, DepthFormer, and IEBins were evaluated, with IEBins emerging as the most suitable model through experimental analysis. The second stage involves training the chosen base model with newly proposed synthetic-based data augmentation techniques and various loss functions to minimize its REL. This stage proves that a combination of Flip, Mask, and CutFlip data augmentation techniques, along with a *SigLoss*-based loss function, yields the most optimal performance. In the third stage, network compression techniques that can minimize the size of the trained base model are identified. Among quantization and pruning methods, FP16-based quantization was proven to be the best combination, optimizing performance in terms of REL, latency, and size. The proposed RMS algorithm’s data augmentation and network compression techniques have enabled significant improvements over the existing IEBins base model: approximately a 4.0% decrease in REL and an 83.4% reduction in model size without any degradation in latency performance. Additionally, the optimal models derived from the RMS algorithm—MonoDepth, DepthFormer, and IEBins—were deployed on the NVIDIA Jetson AGX Orin robot platform [83] for performance analysis. The experimental results indicated that these models are more suitable for static indoor environments when implemented on the NVIDIA Jetson AGX Orin platform. It was also observed that, for more dynamic outdoor environments, a distributed computing approach using multiple NVIDIA Jetson AGX Orin units would be necessary. These results are expected to be highly applicable in real-world scenarios, such as autonomous vehicles and robotics, particularly where data-labeling costs are high and additional data acquisition is challenging. The approach is anticipated to significantly enhance the performance of depth prediction models at a lower cost. While these data augmentation techniques may require significant time investment, it should be noted that this occurs only during the training phase prior to product deployment. For end-users, this approach offers improved REL performance, making it a beneficial strategy for commercialization. Conversely, despite the application of network compression techniques such as pruning and quantization within this study’s RMS algorithm, there are inherent limitations in improving the latency performance of monocular depth estimation. Therefore, it is recommended that future efforts focus on designing a light encoder specifically aimed at achieving low latency.

## Figures and Tables

**Table 1:** Proposed data augmentation techniques proposed for MDE.

	Definition
Mask	The Mask technique employs segmentation technologies to segment
	specific objects, such as cars, from various original images and
	superimposes them onto target original images, thereby synthesizing
	new images.
Mask-Scale	Similar to Mask, segmenting specific objects from various original
	images, adjusting the size and the depth of segmented objects, and then
	superimposing these adjusted segments onto target original images
	to create a new composite.
CutFlip	The left or right half of the image is flipped and copied to the opposite
	side to create a mirrored image. The CutFlip technique allows for three
	variations based on its synthesis method: *CutFlip-L, CutFlip-R, CutFlip-D*.
	·*CutFlip-L* copies the left half of the original image to the right,
	·*CutFlip-R* copies the right half of the original image to the left,
	·*CutFlip-D* swaps and combines the sides of two arbitrary images.

**Table 2:** Parameters for describing RMS.

Notation	Meaning
*B*	Base model of MDE
$W_{B}$	Weights of base model *B*
D	Dataset employed for training
*L*	Loss function employed for training
*Q*	Scheme employed for network compression
$LAT(W_{B})$	Latency of base model *B* with $W_{B}$
$REL(W_{B},D,L)$	REL of MDE model *B* trained with dataset D and by loss function *L*
$\Phi (Q,W_{B^{*}}^{*})$	Compression of $W_{B^{*}}^{*}$ using scheme *Q*
$SIZE(\Phi \left(Q,W_{B^{*}}^{*}\right))$	Size of $\Phi (Q,W_{B^{*}}^{*})$
γ	REL threshold
τ	Latency threshold

**Table 3:** REL results according to data augmentations (Ori.: original dataset, CF: CutFlip, Rot: Rotation, Sca: Scaling, Tr: Translate, Noi: Noise).

	MonoDepth		DepthFormer		IEBins	
Augmentation	SigL	BerhuL	SigL	BerhuL	SigL	BerhuL
Ori.	0.0685	0.0696	0.0528	0.0595	0.0480	0.0487
Ori. + Flip	0.0693	0.0704	0.0534	0.0599	0.0487	0.0494
Ori. + Scale	0.0730	0.0741	0.0570	0.0643	0.0523	0.0531
Ori. + Noise	0.0710	0.0721	0.0551	0.0619	0.0503	0.0512
Ori. + Bright	0.0688	0.0699	0.0528	0.0638	0.0481	0.0489
Ori. + Rotation	0.0689	0.0701	0.0530	0.0597	0.0482	0.0490
Ori. + Mask	0.0696	0.0708	0.0538	0.0612	0.0491	0.0499
Ori. + Mask-Scale	0.0671	0.0681	0.0513	0.0586	0.0466	0.0475
Ori. + Noise+Bright	0.0717	0.0729	0.0559	0.0628	0.0513	0.0521
Ori. + Scale + Bright	0.0719	0.0731	0.0561	0.0632	0.0515	0.0523
Ori. + Flip+Noise	0.0711	0.0722	0.0553	0.0619	0.0506	0.0514
Ori. + Flip + Mask	0.0611	0.0620	0.0510	0.0581	0.0468	0.0476
Ori. + Flip + Mask + CF	0.0610	0.0619	0.0514	0.0571	**0.0461**	0.0469
Ori. + Flip + Mask + Rot + CF	0.0610	0.0620	0.0512	0.0578	0.0465	0.0473
Ori. + Sca + Rot + Tr + Noi + Flip	0.0621	0.0630	0.0519	0.0584	0.0473	0.0480
Ori. + Mask + Mask-Scale + CF	0.0612	0.0622	0.0515	0.0579	0.0463	0.0470
Ori. + Sca + Rot + Tr + Noi + Flip +	0.0616	0.0625	0.0524	0.0565	0.0468	0.0474
Mask + Mask-Scale + CF						

**Table 4:** Latency result according to MDE base models.

	MonoDepth	DepthFormer	IEBins
Latency	24 ms	114 ms	93.3 ms
Size	0.1 GB	3.3 GB	3.3 GB

**Table 5:** Network compression result of the best combination (augmentation and loss) for IEBins.

Scheme	REL	Size (GB)	Latency (ms)
FP32	0.0462	3.3	93.3
FP16	0.0463	0.54	93.8
Pruning (0.05)	0.0463	3.15	96.6
Pruning (0.1)	0.0483	2.99	96.5
Pruning (0.15)	0.0511	2.82	96.1
Pruning (0.1) + FP16	0.0483	0.61	95.9

**Table 6:** Latency results (ms) according to network compression techniques and power consumption.

Latency (ms)	FP32		FP16		FP16 + Pruning	
Power Mode	50 W	30 W	50 W	30 W	50 W	30 W
MonoDepth	87.5	172.2	69.0	143.7	71.1	146.9
DepthFormer	1382.2	2686.4	868.7	2138.5	874.9	2143.8
IEBins	1115.8	2432.8	821.1	1942.7	828.2	1948.3

**Table 7:** Size (GB) according to Network Compression techniques.

Size (GB)	FP32	FP16	FP16 + Pruning
MonoDepth	0.1	0.05	0.06
DepthFormer	3.3	0.56	0.64
IEBins	3.3	0.54	0.61

## Data Availability

Data are contained within the article.
